# First Description of Hypertrophic Cardiomyopathy Phenotype in Apparently Healthy Cats in Morocco: An Echocardiographic Prevalence Study

**DOI:** 10.3390/ani16121802

**Published:** 2026-06-11

**Authors:** Hanaa El Atmani, Faouzi Kichou, Alberto Tarducci, Rahma Azrib, Mohammed Piro

**Affiliations:** 1 Unit of Equines and Small Animals, Department of Medicine, Surgery and Reproduction, Hassan II Agronomic and Veterinary Institute, Rabat BP 6202, Morocco; r.azrib@iav.ac.ma (R.A.); m.piro@iav.ac.ma (M.P.); 2 Unit of Histology and Pathological Anatomy, Department of Veterinary Pathology and Public Health, Hassan II Agronomic and Veterinary Institute, Rabat BP 6202, Morocco; f.kichou@iav.ac.ma; 3 Department of Veterinary Science, University of Turin, 10095 Grugliasco, Italy; alberto.tarducci@unito.it

**Keywords:** heart disease, cardiomyopathy, feline, echocardiography, left ventricular hypertrophy

## Abstract

Hypertrophic Cardiomyopathy Phenotype (HCM-Ph) is the most common group of myocardial disorders in cats. It has been the subject of research worldwide with prevalence rates ranging from 8% to 34%. However, there is no data available on its occurrence in Morocco. This study aimed to investigate the prevalence of HCM-Ph in apparently healthy cats in Morocco, describe the epidemiological and echocardiographic findings of affected cats and to compare patients in ACVIM stages B1 and B2. A total of 81 apparently healthy cats underwent echocardiographic screening. The overall prevalence of HCM-Ph was 9.88% (95% CI: 4.36–18.54%). The median age was four years (IQR 3.5–5.5) and a male overrepresentation (75%) was noted. Affected cats exhibited various echocardiographic patterns of left ventricular hypertrophy, normal left ventricular diameters, preserved fractional shortening and different degrees of left atrial enlargement. Cats in B2 stage were older and had more myocardial hypertrophy compared to cats in B1 stage, highlighting the potential role of wall thickness in clinical staging and risk assessment. Subclinical HCM-Ph is relatively common in apparently healthy cats in Morocco. Veterinarians should be aware of its clinical importance, as despite the fact that it presents no clinical signs, it can have fatal cardiovascular outcomes.

## Simple Summary

Hypertrophic cardiomyopathy phenotype (HCM-Ph) is the most common form of heart muscle disease in cats. It has been the subject of many investigations around the world, with prevalence rates ranging from 8% to 34%. However, it has neither been reported nor described among cats in Morocco. This study aimed to estimate the prevalence of HCM-Ph in apparently healthy cats in Morocco, depict the characteristics of affected cats, and compare patients in low- and high-risk clinical stages. For this purpose, a total of 81 apparently healthy cats underwent cardiac ultrasound examinations. HCM-Ph was found to have a prevalence of 9.88%. A median age of four years and a male predominance were noted. Affected cats had left heart muscle thickening in various portions, normal left ventricular chamber diameters, preserved contractile function and left atrial chamber enlargement. Cats in the high-risk clinical stage were older and had thicker heart muscles than those in the low-risk stage, highlighting the importance of muscle thickness in patient staging. In conclusion, HCM-Ph is relatively common in apparently healthy cats in Morocco. Veterinary practitioners and pet owners should be aware of this, as affected cats may experience life-threatening heart and vascular complications despite having no clinical signs.

## 1. Introduction

Cardiomyopathies (CMPs) are a group of heterogeneous disorders affecting the heart muscle. They include various phenotypic categories that impair myocardial structure and function [1]. In 2020, the American College of Veterinary Internal Medicine (ACVIM) consensus proposed a new classification system for feline CMPs, adapted from the European Society of Cardiology (ESC). This classification system is based on structural and functional features, regardless of the underlying aetiology. In other words, it defines CMP phenotypes, regardless of whether the underlying cause is known [2].

CMPs are the most common heart diseases in cats, with hypertrophic cardiomyopathy being the most prevalent phenotype [3,4,5]. Hypertrophic cardiomyopathy phenotype (HCM-Ph) is characterised by left ventricular hypertrophy (LVH) without chamber dilation. Secondary causes in cats include systemic hypertension, hyperthyroidism, aortic stenosis, acromegaly, neoplasia, transient myocardial thickening (TMT) and dehydration. Infectious myocarditis can also cause secondary LVH in cats, with several agents implicated. The most commonly implicated pathogens are viruses, including feline Coronavirus [6,7], feline immunodeficiency virus [8], and severe acute respiratory syndrome Coronavirus 2 [9,10]. Other infectious agents, including *Toxoplasma gondii* [11,12] and *Bartonella henselae* [13], have also been identified. However, in the majority of LVH cases, no underlying cause can be found; in such cases, the term “hypertrophic cardiomyopathy” (HCM) is used instead [2]. It is worth noting that causative mutations encoding sarcomeric proteins have been identified in Maine Coon, Ragdoll and Sphynx cats [14,15,16]. Several other genetic variants have also been associated with HCM in Bengal cats [17], Domestic Shorthair cats [18,19] and Birman cats [19]. However, a definitive causal link with the disease has yet to be established for these breeds. Most research in the feline literature has focused on HCM, reporting a prevalence of 15% in the general feline population [3,4,20], reaching 29% in older cats [4]. At the same time, several other studies have also addressed HCM-Ph, reporting an overall prevalence ranging from 8% to 34% [20,21,22,23,24].

Cats with HCM-Ph can exhibit a variety of clinical presentations. While most have a subclinical form with no apparent clinical signs, some experience serious clinical manifestations with potentially fatal complications including congestive heart failure (CHF), arterial thromboembolism (ATE) and sudden cardiac death (SCD) [25,26]. Accordingly, affected cats are classified into four ACVIM stages based on their clinical status and echocardiographic findings. Those with the subclinical form are classified in stage B, and can be further subdivided into having a low (B1) or high (B2) risk of developing CHF and/or ATE [2]. Indeed, a large multicentre study has shown that even the subclinical form of HCM negatively impacts cardiac and vascular health over time and increases the risk of cardiovascular morbidity and mortality, thus representing a “global health concern” in cats [26].

Echocardiography is considered the imaging modality of choice for diagnosing HCM-Ph. It plays a pivotal role in phenotype characterisation, prognostic evaluation, patient staging and guiding therapeutic decisions [2,25]. In HCM-Ph, LVH is characterised by its heterogeneity and broad morphological patterns. Indeed, myocardial hypertrophy can affect various left ventricular (LV) segments, ranging from localised to diffuse thickening [27,28]. Therefore, careful investigation of all LV wall segments using both long-axis and short-axis views is required for echocardiographic identification of LVH [2]. Additionally, since regional hypertrophy can be missed by motion mode (M-mode), it is recommended that wall thickness measures be taken on 2D images [2]. Other echocardiographic features of HCM-Ph beside LVH include left atrial enlargement (LAE), left atrial (LA) dysfunction, LV diastolic dysfunction, LV outflow tract obstruction and right ventricular hypertrophy [25,29,30].

While HCM-Ph has been widely subject to research and investigation in many countries around the world, it has neither been reported nor described in Morocco. Currently, no data are available regarding its occurrence and prevalence in apparently healthy cats. Accordingly, the present study aimed to estimate the prevalence of HCM-Ph in apparently healthy cats in Morocco using echocardiographic screening. The study also aimed to describe epidemiological and echocardiographic findings of affected cats and to compare cats in ACVIM stages B1 and B2.

## 2. Materials and Methods

### 2.1. Animals

This cross-sectional study was conducted at the Small Animals Veterinary Teaching Hospital at the Hassan II Agronomic and Veterinary Institute (Rabat, Morocco). Apparently healthy cats owned by clients and presented for a routine examination or annual vaccination were prospectively recruited after obtaining owner consent. Inclusion criteria for enrolment were adult age (over 1 year old) and the absence of current clinical signs and illnesses. Exclusion criteria were a history of systemic disease, current pregnancy and fractious behaviour. The cats were arranged into three age groups [31]: young adults (1–6 years old), mature adults (7–10 years old) and seniors (over 10 years old).

### 2.2. Echocardiographic Examination

The enrolled cats were screened using trans-thoracic echocardiography. Echocardiographic examinations were performed on awake cats, in the lateral recumbency position without sedation, using a 6–10 MHz frequency phased-array transducer and a VINNO G80 VET ultrasound machine (VINNO Technology, Suzhou, China). Two experienced operators (R.A. and H.E.A.) performed all echocardiographic examinations using the same standardised protocol. Right parasternal long-axis and short-axis imaging planes were acquired. Stored echocardiographic images and cine-loops were analysed and measured by a single experienced investigator (H.E.A.).

All echocardiographic measurements were performed on two-dimensional images, and the average of the measurements obtained from three to five consecutive cardiac cycles was recorded as the final value for analysis. End-diastole was identified as the first frame after mitral valve closure in the long-axis plane and as the frame displaying the largest LV chamber dimension in the short-axis plane. End-systole was identified as the frame immediately prior to mitral valve opening in the long-axis view and as the frame displaying the smallest LV chamber dimension in the short-axis view [32].

The thickest LV segments of both the interventricular septum (IVS) and the left ventricular free wall (LVFW) were measured in the long-axis and the short-axis planes; the highest wall thickness value from both views was used as the final measurement for each LV wall (IVSd_max_ and LVFWd_max_). Additionally, the highest wall thickness value between IVSd_max_ and LVFWd_max_ was recorded as LVWTd_max_ for analysis. The leading edge to trailing edge technique was used for IVSd_max_, and the leading edge to leading edge technique (excluding the pericardial sac) was used for LVFWd_max_ [2]. Special consideration was given to ensure that the papillary muscles, chordae tendineae or false tendons were not erroneously included in the measurements. A diagnosis of HCM-Ph was made based on echocardiographic evidence of LVH, which was defined as an IVSd_max_ or LVFWd_max_ of at least 6 mm [2].

The LV end-diastolic (LVIDd) and end-systolic (LVIDs) diameters were measured just below the tips of the mitral valve in the long-axis four-chamber view, using the inner edge to inner edge method [2,32]. To assess LV systolic function, the LV fractional shortening (LV FS) was calculated using the following formula: LV FS% = (LVIDd − LVIDs)/LVIDd × 100.

LA dimensions were measured in both long-axis and short-axis views. In the long-axis view, the maximal end-systolic LA diameter (LADs) was measured in a line parallel to the mitral valve annulus, from the inter-atrial septum to the lateral wall, using the inner edge to inner edge technique [2,32]. In the trans-aortic short-axis view, the left atrial to aortic ratio (LA/AO) was measured in end-systole, which was identified as the first frame after aortic valve closure. This measurement was taken using the inner edge to inner edge method, in the commissure between the non-coronary and the left coronary aortic cusps. Special consideration was given to ensure that the pulmonary veins were not erroneously included in the measurement [33]. LAE was defined as an LA/AO ratio greater than 1.5 or an LAD greater than 16 mm [21,34,35].

According to the ACVIM guidelines, cats with HCM-Ph were classified as stage B1 or B2. Those with normal LA size or mild LAE (LADs ≤ 18 mm or LA/AO ≤ 1.8) were classified as stage B1. Those with moderate-to-severe LAE (LADs > 18 mm or LA/AO > 1.8) were classified as stage B2 [2,25].

### 2.3. Statistical Analysis

Statistical analyses were performed using R software (version 4.5.2). The minimum sample size required to estimate the prevalence of HCM-Ph with a 95% confidence interval (CI) and a precision of ±8% based on an expected prevalence of 15% [4] was *N* = 77 cats. Descriptive statistics were determined for all measured variables. Data normality was assessed using the Shapiro–Wilk test. Categorical variables were presented as frequencies, and continuous data were presented as mean ± standard deviation (SD) or median (interquartile range (IQR)) for normally and non-normally distributed data, respectively. Differences between the groups were assessed using the chi-squared test or Fisher’s exact test for categorical data. For continuous variables, an unpaired Student’s *t* test or Mann–Whitney’s U test was used, depending on normality. To evaluate the possible association between sex and age and the presence of HCM-Ph, a simple binary logistic regression was performed. A *p*-value of less than 0.05 was considered statistically significant.

## 3. Results

A total of 87 apparently healthy cats met the inclusion criteria. Six cats were excluded, as four showed fractious behaviour and two were pregnant. Thus, the study population comprised 81 apparently healthy cats. The characteristics of the entire study population are presented in Table 1. Of these, 44 (54.32%) were female and 37 (45.68%) were male. The median age of cats was three years (IQR 1.5–5). Sixty-nine cats (85.19%) were young adults, seven were mature adults (8.64%), and five were seniors (6.17%). Most of the cats (71) were mixed-breed (87.65%), six were Persian (7.41%), two were Siamese (2.47%), one was a Bengal and one was a British Shorthair (1.23% each).

**Figure 1:**
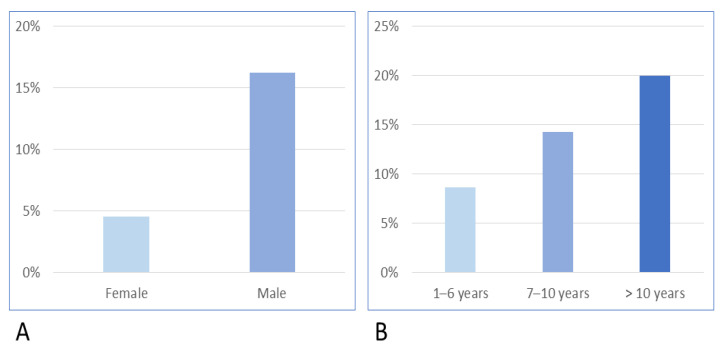
Representation of HCM-Ph prevalence according to sex (**A**) and age group (**B**).

Echocardiographic evidence of LVH was identified in eight cats, giving HCM-Ph an overall prevalence of 9.88% (95% CI: 4.36–18.54%). The remaining 73 cats were classified as normal (90.12%). The prevalence of HCM-Ph was also determined according to sex and age group (Table 2 and Figure 1). The prevalence was found to be 16.22% (95% CI: 6.19–32.01%) in males and 4.55% (95% CI: 0.56–15.47%) in females. The difference between both prevalences was not statistically significant (*p* = 0.133). In young adults, the prevalence was 8.70% (95% CI: 3.26–17.97%). In mature adults, the prevalence was 14.29% (95% CI: 0.36–57.87%). In the senior age group, a prevalence of 20% (95% CI: 0.51–71.64%) was noted. Combining the mature adult and the senior age groups yielded a prevalence of 16.67% (95% CI: 2.09–48.41%). The prevalence of HCM-Ph increased with age, but the difference between the age groups was not statistically significant (*p* = 0.337).

In the simple binary logistic regression, there was no statistically significant association between sex and age and HCM-Ph (Table 3).

**Figure 2:**
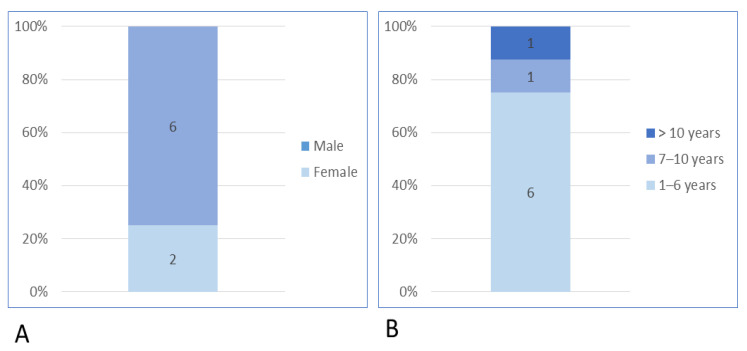
Distribution of cats affected by HCM-Ph by sex (**A**) and age group (**B**).

Of the cats affected by HCM-Ph, six were male (75%) and two were female (25%). Their median age was four years (IQR 3.5–5.5). Six of the cats were young adults, one was a mature adult, and one cat was a senior (Figure 2). Seven of the cats were mixed-breed and one was a Persian.

**Figure 3:**
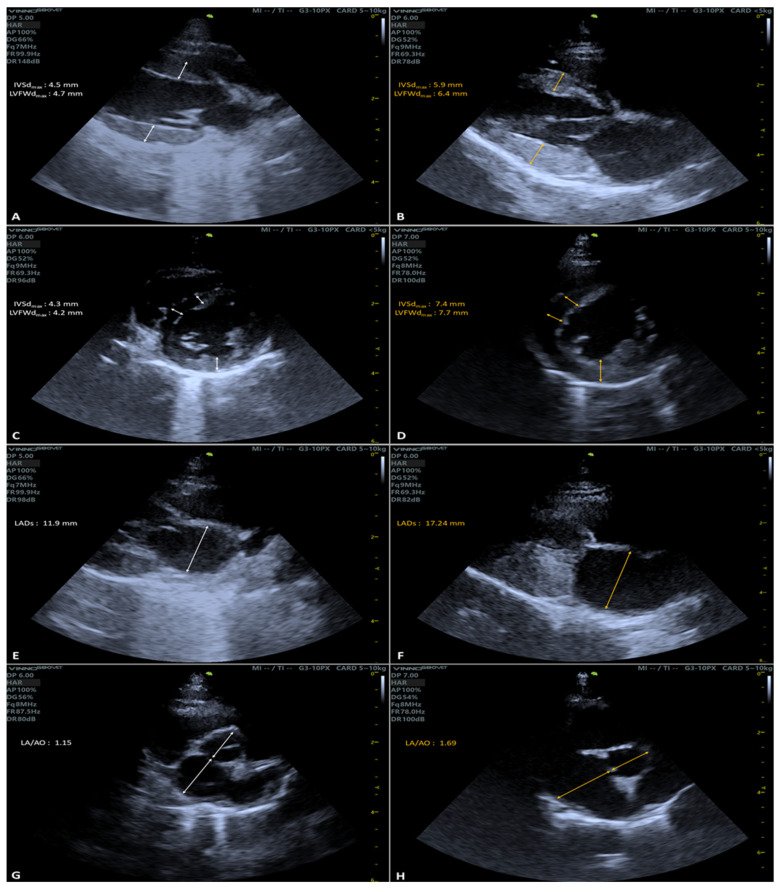
Two-Dimensional echocardiographic long-axis and short-axis images of normal cats and cats with HCM-Ph. Normal LV wall thickness (**A**,**C**) and normal LA size (**E**,**G**). Cats with HCM-Ph with thickened LV walls (**B**,**D**) and enlarged LA (**F**,**H**). HCM-Ph, hypertrophic cardiomyopathy phenotype; LV, left ventricle; LA, left atrium. IVSd_max_, Maximal interventricular septal thickness at end-diastole. LVFWd_max_, Maximal left ventricular free wall thickness at end-diastole. LADs, End-systolic left atrial diameter. LA/AO, Ratio of left atrial dimension to aortic dimension.

Symmetrical hypertrophy affecting the IVS and the LVFW was noted in 50% of cases. Asymmetrical hypertrophy affected the IVS in three cats (37.50%) and affected the LVFW in one cat (12.50%). Four cats (50%) showed evidence of LAE with different degrees; mild LAE was observed in one cat, while moderate-to-severe LAE was seen in three cats. The remaining four cats had normal LA size. Figure 3 shows echocardiographic long-axis and short-axis images of normal cats and cats with HCM-Ph.

Five of the affected cats were classified as stage B1 (62.50%), and three as stage B2 (37.50%). The characteristics and echocardiographic parameters of the B1 and B2 cats, along with the differences between them, are presented in Table 4. Cats in the B2 stage were significantly older than those in the B1 stage, with a median age of seven years (IQR 6–9) compared to four years (IQR 2–4) (*p* = 0.031). Apart from age, there were no differences in signalment between the groups.

**Figure 4:**
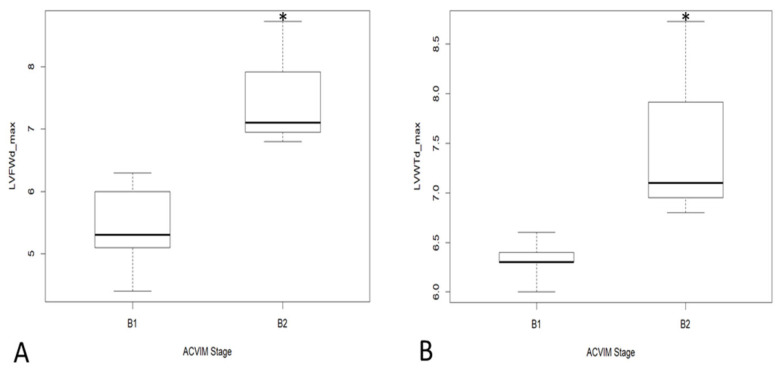
Box-plots of maximal left ventricular free wall thickness (LVFWd_max_) (**A**) and maximal left ventricular wall thickness (LVWTd_max_) (**B**) of HCM-Ph cats in ACVIM stages B1 and B2. * Statistically significant difference (*p* < 0.05).

All LV wall thickness parameters were higher in the B2 group; IVSd_max_ had a mean of 6.47 ± 0.52 mm, LVFWd_max_ had a mean of 7.54 ± 1.04 mm and LVWTd_max_ had a median of 7.10 (IQR 6.95–7.91) mm. In the B1 group, IVSd_max_ had a mean of 6.16 ± 0.43 mm, LVFWd_max_ had a mean of 5.42 ± 0.75 mm and LVWTd_max_ had a median of 6.30 (IQR 6.30–6.40) mm. There was no difference in IVSd_max_ between the groups (*p* = 0.249), whereas both LVFWd_max_ and LVWTd_max_ were statistically significant (*p* = 0.047 and *p* = 0.035, respectively) (Figure 4).

The mean LVIDd and LVIDs for B1 cats were 15.19 ± 1.02 mm and 8.14 ± 0.92 mm, respectively. Meanwhile, B2 cats had a mean LVIDd and LVIDs measurements of 15.87 ± 0.70 mm and 8.20 ± 1.35 mm, respectively. There was no statistical difference in end-diastolic or end-systolic LV dimensions between the groups (*p* = 0.314 and *p* = 0.946, respectively). Regarding LV systolic function, B1 cats had an average LV FS of 46.56% ± 2.87, whereas B2 cats had an average LV FS of 48.44% ± 6.75. The difference between the two groups was not statistically significant (*p* = 0.685).

As LA size was used for group classification, both short-axis and long-axis LA dimensions showed significant differences. B1 cats had a mean LA/AO of 1.28 ± 0.23, and a mean LADs of 14.22 ± 1.53 mm, while B2 cats had significantly higher values, with a mean LA/AO of 1.68 ± 0.01 and a mean LADs of 18.53 ± 0.68 mm (*p* = 0.038 and *p* = 0.0017, respectively).

## 4. Discussion

The present study shows that HCM-Ph is relatively common in apparently healthy cats in Morocco. A primary key point to consider is the difference between HCM and HCM-Ph. The latter is a general term referring to the phenotypic presence of LVH, for both known and unknown causes. In contrast, HCM refers specifically to LVH in the absence of secondary causes, primarily abnormal loading conditions or systemic diseases [2].

In this study, the prevalence of HCM-Ph was 9.88%, which is consistent with previous studies reporting prevalences ranging from 8% to 34% [20,21,22,23,24]. The results demonstrate that HCM-Ph can affect individuals of all ages, with a median age of four years. However, the prevalence curve increased with age, reaching 20% in seniors. The prevalence ratio was 1.4 compared to young adults and 2.2 compared to mature adults. These findings are consistent with those of earlier studies [20,22,23,36]. Payne et al. found in their large-scale study that HCM prevalence reached nearly 30% in seniors, and that increased age was found to be a risk factor for its diagnosis [4]. It is also worth noting that, depending on the cause of LVH, the age at diagnosis may differ. For instance, systemic hypertension and hyperthyroidism typically affect geriatric cats [31], whereas TMT is most frequently observed in young cats [37]. In Maine Coon cats that carry the A31P mutation in the cardiac myosin-binding protein C3 (*MYBPC3*) gene, HCM is most commonly detected at two to three years of age. Late onset at six to seven years of age has also been reported; however, it is less frequent [14]. In homozygous-affected cats, the penetrance appears to increase with age [38]. In contrast, HCM develops much earlier in Ragdoll cats with the *MYBPC3* R820W mutation, with a mean age of onset of 15 to 21 months [15].

Male predominance was also noted, along with a prevalence 3.5 times higher compared to females. This finding is supported by numerous studies which have also demonstrated male overrepresentation in HCM-Ph [4,21,22,39,40]. Furthermore, male sex has been identified as a risk factor for HCM, with an odds ratio of up to 7.89 compared to females [4,34]. Additionally, male Maine Coon cats with the genetic form of HCM are significantly more affected than female cats [38] and show earlier disease onset with quicker progression to severe forms [41]. These observations are likely attributable to environmental factors, hormones and modifier genes [4,40,41]. Despite the relatively higher prevalence, neither sex nor age were associated with HCM-Ph in the logistic regression analysis, which is likely due to the relatively small sample size of the study. As the majority of the cats studied were mixed-breed, no explicit conclusions could be drawn about breed predisposition.

Echocardiographic evidence of hypertrophy was present in various segments of both the IVS and the LVFW, highlighting the heterogeneity of HCM-Ph. The most commonly encountered pattern was symmetrical LVH, consistent with most studies [27,34,37]. However, it is worth noting that, while HCM in humans mostly involves the IVS [42], studies in cats seem to vary [36,39,43]. This inconsistency may be due to the different measurement locations and different definition criteria used in feline cardiology [23,28,34]. Conversely, it could be argued that, since each cause of LVH has its own underlying pathophysiological mechanism, the morphological pattern may vary accordingly. However, one feline study found no explicit differentiation between HCM and secondary hypertrophy, suggesting that no echocardiographic pattern is typical of any LVH aetiology [44]. Additionally, some authors have suggested that cat breed and population may also play a role [28,34], but more studies are needed to confirm this. Overall, these findings emphasise the variable morphological expression of HCM-Ph, regardless of the underlying mechanism.

The results of this study showed that the LV diameters of both B1 and B2 cats fell within the reference interval, indicating normal values. This is consistent with the concentric type of thickening in HCM-Ph, which is characterised by normal or occasionally decreased LV chamber dimensions [25]. In HCM, chamber size is independently associated with wall thickness [39], and a smaller LVIDd is associated with an increased risk of developing the disease [29] and is also an independent predictor of diagnosis [45]. In secondary LVH, concentric hypertrophy is also typically present, although evidence of LV enlargement has occasionally been described [44,46,47]. Similarly, adverse remodelling with chamber dilation and wall thinning can also occur in end-stage HCM [46,47].

The affected cats had a normal LV FS, which corroborates the preserved overall systolic function observed in HCM-Ph [25]. However, substantial systolic dysfunction with reduced LV FS can also be noted in advanced end-stage HCM and in some hyperthyroid cats [46,47,48].

When comparing B1 and B2 cats, except for the LA size measures used for classification, there were significant differences in age, LVFWd_max_ and LVWTd_max_ between the groups. B2 cats were significantly older than B1 cats. Theoretically, this finding implies that older HCM-Ph patients are at a higher risk of developing cardiovascular morbidity. However, this inference cannot be made due to the cross-sectional design of the study. Nevertheless, it has been well established by many research investigations [26,35,49,50]. Indeed, the REVEAL study showed a substantially increased risk of CHF, ATE and cardiovascular death in HCM cats with increasing age [26]. Furthermore, numerous studies have reported older age as an independent predictor of poor prognosis [49,50]. On the other hand, a breed effect may also be involved. Certain breeds, mainly Maine Coon and Ragdoll cats, exhibit an earlier onset of cardiovascular morbidity and mortality, even before reaching one year of age. This is particularly evident in homozygous cats for the mutation, but also surprisingly in the wild-type status as well [28,50,51].

The increased LVFWd_max_ and LVWTd_max_ observed in B2 cats indicate greater hypertrophy than in B1 cats. Indeed, myocardial hypertrophy tends to be greater in more advanced stages [49], and even in the subclinical form, B2 patients have thicker walls than B1 patients [52]. These findings can be partly explained by the correlation between LA size and LV wall thickness [30], which in turn correlates with the underlying myocardial impairment. Firstly, in the context of HCM, greater LV thickening is associated with greater structural alterations and myocardial dysfunction [53]. Although dysfunction can be observed in non-hypertrophied segments, studies have demonstrated a correlation between wall thickness and dysfunction [54,55]. Secondly, in feline hyperthyroidism, greater LVH and LAE are observed in cats with higher thyroxine concentrations [56], as the cardiovascular system is under much greater strain. Similarly, in humans with hypertension, both systolic and diastolic blood pressure are associated with increased hypertrophy [57], but no such correlation is found in hypertensive cats [58]. This suggests the possible involvement of additional species-specific factors and highlights the need for further studies. On the other hand, both LA size and LV wall thickness are markers of clinical morbidity and severity in HCM. In fact, research has shown that significant LVH and LAE are associated with a poor prognosis and with a shorter survival [27,43,44]. In one study, severe hypertrophy, defined by a wall thickness of more than 9 mm, was an independent predictor of an increased risk of cardiac death [35], a finding that has previously been reported in human HCM [59]. Taken together, these observations highlight the potential role of LV wall thickness in staging and risk assessment, particularly in equivocal cases involving borderline LA size values or where there is conflicting evidence regarding the severity of LAE. Indeed, severe LVH has been proposed as an additional factor in ACVIM staging [2], but a standardised cut-off point has yet to be determined. The limited sample size of this study hindered the analysis of receiver operating characteristics (ROC), so future investigations are needed to evaluate the diagnostic performance of wall thickness in clinical staging, and to determine reliable optimal cut-offs.

Only HCM-Ph was encountered in the studied population; other CMPs were not identified. This can be partly explained by its higher prevalence compared to other phenotypes, as previously reported [5]. Also, unlike the common subclinical form of HCM-Ph, other heart diseases often have symptomatic presentations [2,21].

To the authors’ knowledge, this is the first report describing feline HCM-Ph and its characteristics in Morocco, indicating its relatively common occurrence in approximately one in ten apparently healthy cats. Although the affected cats presented no apparent clinical signs, the practical relevance of subclinical HCM-Ph lies in its substantial clinical consequences. Indeed, HCM is considered a “global health concern” and the main cause of cardiovascular morbidity and mortality in cats [26]. Large multicentre studies have shown that while some patients can enjoy an uneventful, normal life expectancy, approximately one-third can experience at least one cardiovascular event (CHF, ATE or SCD) [26,29,60]. Additionally, due to the burden of the disease, these patients have a much higher overall mortality rate than healthy cats [61]. Cardiovascular outcomes in secondary LVH are less frequently studied, probably due to their rarity. However, several case series have reported evidence of CHF [48,62,63,64,65], ATE [66] and SCD [64]. It is worth mentioning that an antecedent event can be identified in nearly half of HCM-Ph cats presenting with CHF, including stress, anaesthesia, surgery, intravenous fluid therapy and administrations of corticosteroids [37,49]. These events are thought to precipitate decompensation through plasma volume expansion [67] and other mechanisms that have yet to be determined. Accordingly, the importance of subclinical HCM-Ph detection cannot be overemphasised. Cardiac investigation is therefore particularly recommended for cats with suspicious physical examination findings, HCM mutation carriers, relatives of affected patients, pedigree cats intended for breeding, endocrine patients and old cats intended for “CHF precipitating” interventions [2].

The results reported herein should be considered in light of the following limitations. Firstly, although the minimum sample size was determined by precision analysis, the number of cats studied was relatively small, thereby widening the prevalence confidence interval. Additionally, the small size of the study sample limits the reliability of normality testing and the statistical power of group comparisons. Therefore, the study results should be interpreted with caution. However, it is important to note that none of the variables that reached statistical significance showed any data outliers, suggesting that the *p*-values and the observed effects are not influenced by extreme observations. Nevertheless, further studies with larger samples and greater statistical power are needed to confirm prevalence and ensure generalisability. Secondly, due to the cross-sectional design of the study, it was not possible to follow up with patients. Consequently, it was not possible to monitor myocardial thickening over time. This precluded the detection of reversible and transient forms of LVH. Blood pressure measurement, quantification of serum thyroxine levels, and cardiac biomarker testing were also not carried out. Consequently, it was not possible to differentiate primary HCM from secondary LVH, including that caused by systemic hypertension, hyperthyroidism and myocarditis. However, the focus of this study was on the overall echocardiographic phenotype rather than the underlying aetiologies. Thus, the term “HCM-Ph” was used, without implying a definitive diagnosis of the underlying cause of LVH. Therefore, based on the study findings, it must be emphasised that inferences regarding the aetiology of myocardial hypertrophy cannot be drawn. Accordingly, further studies are needed to assess the prevalence of idiopathic HCM, hyperthyroidism, and systemic hypertension in cats in Morocco. Furthermore, investigating the genetic background, dietary regimens, and lifestyle characteristics of cats would be highly informative. Such investigations would provide valuable insights into the epidemiological features of both HCM and secondary LVH in feline populations in Morocco. Thirdly, as the study took place in a teaching referral hospital, the results may not be representative of the general cat population. However, only apparently healthy cats that presented spontaneously for a routine examination were enrolled. Additionally, the inter-operator variability for obtaining the echocardiographic images was not performed; however, both operators used the same standardised protocol, and all the measurements and the interpretations were performed by an operator with good repeatability. Moreover, a concurrent ECG tracing was unavailable during the echocardiographic examinations. However, the identification of the cardiac cycle phases was based on reliable mechanical indicators, mainly valve movements and cardiac dimensions [32]. Furthermore, the echocardiographic assessment of diastolic function was not performed; however, it is not required for diagnosing HCM-Ph and determining its prevalence. Further prospective studies are warranted to assess LV diastolic performance, LA function, LA appendage flow and overall cardiovascular haemodynamics. These parameters would enable a more thorough echocardiographic characterisation of feline HCM-Ph in Morocco. Finally, this study focused solely on cardiac ultrasound, which is the gold standard for diagnosing CMPs. However, findings from heart auscultation and other diagnostic modalities, such as electrocardiography and thoracic radiography, would have been more informative.

## 5. Conclusions

This study is the first to report the presence of HCM-Ph in apparently healthy cats in Morocco, with a prevalence of 9.88%. HCM-Ph was found in cats of all ages and sexes, but a higher prevalence was noted in males and older cats. Affected cats exhibited concentric LVH with various echocardiographic patterns, normal LV chamber dimensions, preserved overall systolic function and different degrees of LAE. Cats in stage B2 were older and had thicker LV walls compared to stage B1, highlighting the potential role of wall thickness along with LA size measures in patient staging. Veterinary practitioners should be aware of the clinical importance of subclinical HCM-Ph, as despite presenting no apparent clinical signs, it can have serious, life-threatening outcomes. Further larger studies are required to explore the diagnostic performance of additional echocardiographic parameters and to determine reliable cut-offs for risk assessment and clinical decision-making.

## Figures and Tables

**Table 1:** Signalment of overall study population.

Signalment	Overall Population
Male Female	37 (45.68%) 44 (54.32%)
Age (years)	3 (IQR 1.5–5)
Young adults (1–6 years) Mature adults (7–10 years) Seniors (>10 years)	69 (85.19%) 7 (8.64%) 5 (6.17%)
Mixed-breed Persian Siamese Bengal British Shorthair	71 (87.65%) 6 (7.41%) 2 (2.47%) 1 (1.23%) 1 (1.23%)

**Table 2:** Prevalence of HCM-Ph in overall population and based on sex and age group.

Group	HCM-Ph Prevalence	*p*-Value
Overall population	9.88%	-
Male Female	16.22% 4.55%	0.133
Young adults (1–6 years) Mature adults (7–10 years) Seniors (>10 years)	8.70% 14.29% 20.00%	0.337

**Table 3:** Results of the simple binary logistic regression for the presence of HCM-Ph.

Parameter	Odds Ratio (95% CI)	*p*-Value
Sex	4.06 (0.87–29.04)	0.099
Age	1.12 (0.89–1.37)	0.274

**Table 4:** Signalment and echocardiographic variables of HCM-Ph cats in ACVIM stages B1 and B2.

Parameters	ACVIM B1	ACVIM B2	*p*-Value
Male Female	3 2	3 0	0.464
Age (years)	4 (IQR 2–4)	7 (IQR 6–9)	0.031 *
Young adults (1–6 years) Mature adults (7–10 years) Seniors (>10 years)	5 - -	1 1 1	0.107
Mixed-breed Persian	4 1	3 -	1
IVSd_max_ (mm)	6.16 ± 0.43	6.47 ± 0.52	0.249
LVFWd_max_ (mm)	5.42 ± 0.75	7.54 ± 1.04	0.047 *
LVWTd_max_ (mm)	6.30 (IQR 6.30–6.40)	7.10 (IQR 6.95–7.91)	0.035 *
LVIDd (mm)	15.19 ± 1.02	15.87 ± 0.70	0.314
LVIDs (mm)	8.14 ± 0.92	8.20 ± 1.35	0.946
LV FS (%)	46.56 ± 2.87	48.44 ± 6.75	0.685
LA/AO	1.28 ± 0.23	1.68 ± 0.01	0.038 *
LADs (mm)	14.22 ± 1.53	18.53 ± 0.68	0.0017 *

* Statistically significant difference (*p* < 0.05).

## Data Availability

Research data are not currently available due to ongoing research.

## Abbreviations

The following abbreviations are used in this manuscript:

HCM-Ph	Hypertrophic Cardiomyopathy Phenotype
CMP	Cardiomyopathy
ACVIM	American College of Veterinary Internal Medicine
ESC	European Society of Cardiology
LVH	Left Ventricular Hypertrophy
TMT	Transient Myocardial Thickening
HCM	Hypertrophic Cardiomyopathy
CHF	Congestive Heart Failure
ATE	Arterial Thromboembolism
SCD	Sudden Cardiac Death
LV	Left Ventricle
LAE	Left Atrial Enlargement
LA	Left Atrium
IVS	Interventricular Septum
LVFW	Left Ventricular Free Wall
IVSd_max_	Maximal Interventricular Septal thickness at end-Diastole
LVFWd_max_	Maximal Left Ventricular Free Wall thickness at end-Diastole
LVWTd_max_	Maximal Left Ventricular Wall Thickness at end-Diastole
LVIDd	Left Ventricular Internal Diameter at end-Diastole
LVIDs	Left Ventricular Internal Diameter at end-Systole
LV FS	Left Ventricular Fractional Shortening
LADs	Left Atrial Diameter at end-Systole
LA/AO	Ratio of Left Atrial dimension to Aortic dimension
SD	Standard Deviation
IQR	Interquartile Range
CI	Confidence Interval
MYBPC3	Cardiac Myosin-Binding Protein C3
ROC	Receiver Operating Characteristics

## References

[1] Elliott P., Andersson B., Arbustini E., Bilinska Z., Cecchi F., Charron P., Dubourg O., Kühl U., Maisch B., McKenna W.J. (2008). Classification of the Cardiomyopathies: A Position Statement from the European Society of Cardiology Working Group on Myocardial and Pericardial Diseases. Eur. Heart J..

[2] Luis Fuentes V., Abbott J., Chetboul V., Côté E., Fox P.R., Häggström J., Kittleson M.D., Schober K., Stern J.A. (2020). ACVIM Consensus Statement Guidelines for the Classification, Diagnosis, and Management of Cardiomyopathies in Cats. J. Vet. Intern. Med..

[3] Paige C.F., Abbott J.A., Elvinger F., Pyle R.L. (2009). Prevalence of Cardiomyopathy in Apparently Healthy Cats. J. Am. Vet. Med. Assoc..

[4] Payne J.R., Brodbelt D.C., Luis Fuentes V. (2015). Cardiomyopathy Prevalence in 780 Apparently Healthy Cats in Rehoming Centres (the CatScan Study). J. Vet. Cardiol..

[5] Ferasin L., Sturgess C.P., Cannon M.J., Caney S.M.A., Gruffydd-Jones T.J., Wotton P.R. (2003). Feline Idiopathic Cardiomyopathy: A Retrospective Study of 106 Cats (1994–2001). J. Feline Med. Surg..

[6] Ernandes M.A., Cantoni A.M., Armando F., Corradi A., Ressel L., Tamborini A. (2019). Feline Coronavirus-Associated Myocarditis in a Domestic Longhair Cat. J. Feline Med. Surg. Open Rep..

[7] Korzybska E., O’Halloran C., Culshaw G., Milevoj N., Fernandez-Gallego A., Oliveira M.I. (2025). Successful Treatment of Feline Infectious Peritonitis-Associated Myocarditis in a Cat. J. Feline Med. Surg. Open Rep..

[8] Rolim V.M., Casagrande R.A., Wouters A.T.B., Driemeier D., Pavarini S.P. (2016). Myocarditis Caused by Feline Immunodeficiency Virus in Five Cats with Hypertrophic Cardiomyopathy. J. Comp. Pathol..

[9] Ferasin L., Fritz M., Ferasin H., Becquart P., Corbet S., Ar Gouilh M., Legros V., Leroy E.M. (2021). Infection with SARS-CoV-2 Variant B.1.1.7 Detected in a Group of Dogs and Cats with Suspected Myocarditis. Vet. Rec..

[10] Chetboul V., Foulex P., Kartout K., Klein A.M., Sailleau C., Dumarest M., Delaplace M., Ar Gouilh M., Mortier J., Le Poder S. (2021). Myocarditis and Subclinical-Like Infection Associated with SARS-CoV-2 in Two Cats Living in the Same Household in France: A Case Report with Literature Review. Front. Vet. Sci..

[11] Romito G., Fracassi F., Cipone M. (2022). Transient Myocardial Thickening Associated with Acute Myocardial Injury and Congestive Heart Failure in Two *Toxoplasma Gondii* -Positive Cats. J. Feline Med. Surg. Open Rep..

[12] Simpson K.E., Devine B.C., Gunn-Moore D. (2005). Suspected Toxoplasma—Associated Myocarditis in a Cat. J. Feline Med. Surg..

[13] Joseph J.L., Oxford E.M., Santilli R.A. (2018). Transient Myocardial Thickening in a *Bartonella Henselae*–Positive Cat. J. Vet. Cardiol..

[14] Meurs K.M., Sanchez X., David R.M., Bowles N.E., Towbin J.A., Reiser P.J., Kittleson J.A., Munro M.J., Dryburgh K., MacDonald K.A. (2005). A Cardiac Myosin Binding Protein C Mutation in the Maine Coon Cat with Familial Hypertrophic Cardiomyopathy. Hum. Mol. Genet..

[15] Meurs K.M., Norgard M.M., Ederer M.M., Hendrix K.P., Kittleson M.D. (2007). A Substitution Mutation in the Myosin Binding Protein C Gene in Ragdoll Hypertrophic Cardiomyopathy. Genomics.

[16] Meurs K.M., Williams B.G., DeProspero D., Friedenberg S.G., Malarkey D.E., Ezzell J.A., Keene B.W., Adin D.B., DeFrancesco T.C., Tou S. (2021). A Deleterious Mutation in the ALMS1 Gene in a Naturally Occurring Model of Hypertrophic Cardiomyopathy in the Sphynx Cat. Orphanet J. Rare Dis..

[17] Demeekul K., Sukumolanan P., Panprom C., Thaisakun S., Roytrakul S., Petchdee S. (2022). Echocardiography and MALDI-TOF Identification of Myosin-Binding Protein C3 A74T Gene Mutations Involved Healthy and Mutated Bengal Cats. Animals.

[18] Schipper T., Van Poucke M., Sonck L., Smets P., Ducatelle R., Broeckx B.J.G., Peelman L.J. (2019). A Feline Orthologue of the Human *MYH7* c.5647G>A (p.(Glu1883Lys)) Variant Causes Hypertrophic Cardiomyopathy in a Domestic Shorthair Cat. Eur. J. Hum. Genet..

[19] Raffle J., Novo Matos J., Wallace M., Wilkie L., Piercy R.J., Elliott P., Connolly D.J., Luis Fuentes V., Psifidi A. (2025). Identification of Novel Genetic Variants Associated with Feline Cardiomyopathy Using Targeted Next-Generation Sequencing. Sci. Rep..

[20] Seo J., Owen R., Hunt H., Luis Fuentes V., Connolly D.J., Munday J.S. (2025). Prevalence of Cardiomyopathy and Cardiac Mortality in a Colony of Non-Purebred Cats in New Zealand. N. Z. Vet. J..

[21] Wagner T., Luis Fuentes V., Payne J.R., McDermott N., Brodbelt D. (2010). Comparison of Auscultatory and Echocardiographic Findings in Healthy Adult Cats. J. Vet. Cardiol..

[22] Riesen S.C., Kovacevic A., Lombard C.W., Amberger C. (2007). Echocardiographic Screening of Purebred Cats: An Overview from 2002 to 2005. Schweiz. Arch. Tierheilk..

[23] Gundler S., Tidholm A., Häggström J. (2008). Prevalence of Myocardial Hypertrophy in a Population of Asymptomatic Swedish Maine Coon Cats. Acta Vet. Scand..

[24] Park S.-W., Kim K., Lee Y.-J., Do Y.-J., Ro W.-B., Lee C.-M. (2025). Prevalence and Characteristics of Transient Myocardial Thickening in Cats with Hypertrophic Cardiomyopathy Phenotypes. Vet. Q..

[25] Kittleson M.D., Côté E. (2021). The Feline Cardiomyopathies: 2. Hypertrophic Cardiomyopathy. J. Feline Med. Surg..

[26] Fox P.R., Keene B.W., Lamb K., Schober K.A., Chetboul V., Luis Fuentes V., Wess G., Payne J.R., Hogan D.F., Motsinger-Reif A. (2018). International Collaborative Study to Assess Cardiovascular Risk and Evaluate Long-term Health in Cats with Preclinical Hypertrophic Cardiomyopathy and Apparently Healthy Cats: The REVEAL Study. J. Vet. Intern. Med..

[27] Brizard D., Amberger C., Hartnack S., Doherr G.M., Lombard C. (2009). Phenotypes and Echocardiographic Characteristics of a European Population of Domestic Shorthair Cats with Idiopathic Hypertrophic Cardiomyopathy. Schweiz. Arch. Tierheilk..

[28] Trehiou-Sechi E., Tissier R., Gouni V., Misbach C., Petit A.M.P., Balouka D., Carlos Sampedrano C., Castaignet M., Pouchelon J.-L., Chetboul V. (2012). Comparative Echocardiographic and Clinical Features of Hypertrophic Cardiomyopathy in 5 Breeds of Cats: A Retrospective Analysis of 344 Cases (2001–2011). J. Vet. Intern. Med..

[29] Novo Matos J., Payne J.R., Seo J., Luis Fuentes V. (2022). Natural History of Hypertrophic Cardiomyopathy in Cats from Rehoming Centers: The CatScan II Study. J. Vet. Intern. Med..

[30] Visser L.C., Sloan C.Q., Stern J.A. (2017). Echocardiographic Assessment of Right Ventricular Size and Function in Cats with Hypertrophic Cardiomyopathy. J. Vet. Intern. Med..

[31] Quimby J., Gowland S., Carney H.C., DePorter T., Plummer P., Westropp J. (2021). 2021 AAHA/AAFP Feline Life Stage Guidelines. J. Feline Med. Surg..

[32] Lang R.M., Badano L.P., Mor-Avi V., Afilalo J., Armstrong A., Ernande L., Flachskampf F.A., Foster E., Goldstein S.A., Kuznetsova T. (2015). Recommendations for Cardiac Chamber Quantification by Echocardiography in Adults: An Update from the American Society of Echocardiography and the European Association of Cardiovascular Imaging. J. Am. Soc. Echocardiogr..

[33] Hansson K., Häggström J., Kvart C., Lord P. (2002). Left Atrial to Aortic Root Indices Using Two-Dimensional and M-Mode Echocardiography in Cavalier King Charles Spaniels with and without Left Atrial Enlargement. Vet. Radiol. Ultrasound.

[34] Granström S., Nyberg Godiksen M.T., Christiansen M., Pipper C.B., Willesen J.T., Koch J. (2011). Prevalence of Hypertrophic Cardiomyopathy in a Cohort of British Shorthair Cats in Denmark. J. Vet. Intern. Med..

[35] Payne J.R., Borgeat K., Connolly D.J., Boswood A., Dennis S., Wagner T., Menaut P., Maerz I., Evans D., Simons V.E. (2013). Prognostic Indicators in Cats with Hypertrophic Cardiomyopathy. J. Vet. Intern. Med..

[36] Chetboul V., Petit A., Gouni V., Trehiou-Sechi E., Misbach C., Balouka D., Carlos Sampedrano C., Pouchelon J.-L., Tissier R., Abitbol M. (2012). Prospective Echocardiographic and Tissue Doppler Screening of a Large Sphynx Cat Population: Reference Ranges, Heart Disease Prevalence and Genetic Aspects. J. Vet. Cardiol..

[37] Novo Matos J., Pereira N., Glaus T., Wilkie L., Borgeat K., Loureiro J., Silva J., Law V., Kranjc A., Connolly D.J. (2018). Transient Myocardial Thickening in Cats Associated with Heart Failure. J. Vet. Intern. Med..

[38] Longeri M., Ferrari P., Knafelz P., Mezzelani A., Marabotti A., Milanesi L., Pertica G., Polli M., Brambilla P.G., Kittleson M. (2013). *Myosin-Binding Protein C* DNA Variants in Domestic Cats (A31P, A74T, R820W) and Their Association with Hypertrophic Cardiomyopathy. J. Vet. Intern. Med..

[39] Seo J., Loh Y., Connolly D.J., Luis Fuentes V., Dutton E., Hunt H., Munday J.S. (2024). Prevalence of Hypertrophic Cardiomyopathy and ALMS1 Variant in Sphynx Cats in New Zealand. Animals.

[40] Mary J., Chetboul V., Sampedrano C.C., Abitbol M., Gouni V., Trehiou-Sechi E., Tissier R., Queney G., Pouchelon J.-L., Thomas A. (2010). Prevalence of the MYBPC3-A31P Mutation in a Large European Feline Population and Association with Hypertrophic Cardiomyopathy in the Maine Coon Breed. J. Vet. Cardiol..

[41] Kittleson M.D., Meurs K.M., Munro M.J., Kittleson J.A., Liu S.-K., Pion P.D., Towbin J.A. (1999). Familial Hypertrophic Cardiomyopathy in Maine Coon Cats: An Animal Model of Human Disease. Circulation.

[42] Klues H.G., Schiffers A., Maron B.J. (1995). Phenotypic Spectrum and Patterns of Left Ventricular Hypertrophy in Hypertrophic Cardiomyopathy: Morphologic Observations and Significance as Assessed by Two-Dimensional Echocardiography in 600 Patients. J. Am. Coll. Cardiol..

[43] Fox P.R., Liu S.-K., Maron B.J. (1995). Echocardiographic Assessment of Spontaneously Occurring Feline Hypertrophic Cardiomyopathy: An Animal Model of Human Disease. Circulation.

[44] Spalla I., Locatelli C., Riscazzi G., Santagostino S., Cremaschi E., Brambilla P. (2015). Survival in Cats with Primary and Secondary Cardiomyopathies. J. Feline Med. Surg..

[45] Spalla I., Boswood A., Connolly D.J., Luis Fuentes V. (2019). Speckle Tracking Echocardiography in Cats with Preclinical Hypertrophic Cardiomyopathy. J. Vet. Intern. Med..

[46] Baty C.J., Malarkey D.E., Atkins C.E., DeFrancesco T.C., Sidley J., Keene B.W. (2001). Natural History of Hypertrophic Cardiomyopathy and Aortic Thromboembolism in a Family of Domestic Shorthair Cats. J. Vet. Intern. Med..

[47] Cesta M.F., Baty C.J., Keene B.W., Smoak I.W., Malarkey D.E. (2005). Pathology of End-Stage Remodeling in a Family of Cats with Hypertrophic Cardiomyopathy. Vet. Pathol..

[48] Jacobs G., Hutson C., Dougherty J., Kirmayer A. (1986). Congestive Heart Failure Associated with Hyperthyroidism in Cats. J. Am. Vet. Med. Assoc..

[49] Rush J.E., Freeman L.M., Fenollosa N.K., Brown D.J. (2002). Population and Survival Characteristics of Cats with Hypertrophic Cardiomyopathy: 260 Cases (1990–1999). J. Am. Vet. Med. Assoc..

[50] Payne J., Luis Fuentes V., Boswood A., Connolly D., Koffas H., Brodbelt D. (2010). Population Characteristics and Survival in 127 Referred Cats with Hypertrophic Cardiomyopathy (1997 to 2005). J. Small Anim. Pract..

[51] Kittelson M.D., Meurs K.M., Harris S.P. (2015). The Genetic Basis of Hypertrophic Cardiomyopathy in Cats and Humans. J. Vet. Cardiol..

[52] Li Q., Homilius M., Achilles E., Massey L.K., Convey V., Ohlsson Å., Ljungvall I., Häggström J., Boler B.V., Steiner P. (2025). Metabolic Abnormalities and Reprogramming in Cats with Naturally Occurring Hypertrophic Cardiomyopathy. ESC Heart Fail..

[53] Popovic Z.B., Kwon D.H., Mishra M., Buakhamsri A., Greenberg N.L., Thamilarasan M., Flamm S.D., Thomas J.D., Lever H.M., Desai M.Y. (2008). Association between Regional Ventricular Function and Myocardial Fibrosis in Hypertrophic Cardiomyopathy Assessed by Speckle Tracking Echocardiography and Delayed Hyperenhancement Magnetic Resonance Imaging. J. Am. Soc. Echocardiogr..

[54] Wess G., Sarkar R., Hartmann K. (2010). Assessment of Left Ventricular Systolic Function by Strain Imaging Echocardiography in Various Stages of Feline Hypertrophic Cardiomyopathy. J. Vet. Intern. Med..

[55] Suzuki R., Mochizuki Y., Yoshimatsu H., Teshima T., Matsumoto H., Koyama H. (2017). Determination of Multidirectional Myocardial Deformations in Cats with Hypertrophic Cardiomyopathy by Using Two-Dimensional Speckle-Tracking Echocardiography. J. Feline Med. Surg..

[56] Watson N., Murray J.K., Fonfara S., Hibbert A. (2018). Clinicopathological Features and Comorbidities of Cats with Mild, Moderate or Severe Hyperthyroidism: A Radioiodine Referral Population. J. Feline Med. Surg..

[57] Nikorowitsch J., Bei der Kellen R., Haack A., Magnussen C., Prochaska J., Wild P.S., Dörr M., Twerenbold R., Schnabel R.B., Kirchhof P. (2023). Correlation of Systolic and Diastolic Blood Pressure with Echocardiographic Phenotypes of Cardiac Structure and Function from Three German Population-Based Studies. Sci. Rep..

[58] Chetboul V., Lefebvre H.P., Pinhas C., Clerc B., Boussouf M., Pouchelon J.-L. (2003). Spontaneous Feline Hypertension: Clinical and Echocardiographic Abnormalities, and Survival Rate. J. Vet. Intern. Med..

[59] Spirito P., Bellone P., Harris K.M., Bernabo P., Bruzzi P., Maron B.J. (2000). Magnitude of Left Ventricular Hypertrophy and Risk of Sudden Death in Hypertrophic Cardiomyopathy. N. Engl. J. Med..

[60] Ironside V.A., Tricklebank P.R., Boswood A. (2021). Risk Indictors in Cats with Preclinical Hypertrophic Cardiomyopathy: A Prospective Cohort Study. J. Feline Med. Surg..

[61] Fox P.R., Keene B.W., Lamb K., Schober K.E., Chetboul V., Luis Fuentes V., Payne J.R., Wess G., Hogan D.F., Abbott J.A. (2019). Long-term Incidence and Risk of Noncardiovascular and All-cause Mortality in Apparently Healthy Cats and Cats with Preclinical Hypertrophic Cardiomyopathy. J. Vet. Intern. Med..

[62] Johnson J., Melhorn H., Karchemskiy S., Karlin E., Bain P., Rush J., Peterson C. (2024). Case Report: Diffuse Large B-Cell Lymphoma Presenting as Congestive Heart Failure in a Cat. Front. Vet. Sci..

[63] Peterson M.E., Taylor R.S., Greco D.S., Nelson R.W., Randolph J.F., Foodman M.S., Moroff S.D., Morrison S.A., Lothrop C.D. (1990). Acromegaly in 14 Cats. J. Vet. Intern. Med..

[64] Elliott J., Barber P.J., Syme H.M., Rawlings J.M., Markwell P.J. (2001). Feline Hypertension: Clinical Findings and Response to Antihypertensive Treatment in 30 Cases. J. Small Anim. Pract..

[65] Wey A.C., Atkins C.E. (2000). Aortic Dissection and Congestive Heart Failure Associated with Systemic Hypertension in a Cat. J. Vet. Intern. Med..

[66] Vollmar C., Mitropoulou A., Hassdenteufel E., Hildebrandt N., Schneider M. (2024). Arterial Thromboembolism in a Cat with Transient Myocardial Thickening. J. Vet. Cardiol..

[67] Block C.L., Oyama M.A. (2020). Echocardiographic and Biomarker Evidence of Plasma Volume Expansion after Short-term Steroids Administered Orally in Cats. J. Vet. Intern. Med..

